# Engineered exosomes enriched with select microRNAs amplify their therapeutic efficacy for traumatic brain injury and stroke

**DOI:** 10.3389/fncel.2024.1376601

**Published:** 2024-03-19

**Authors:** Liang Chen, Ye Xiong, Michael Chopp, Yanlu Zhang

**Affiliations:** ^1^Department of Neurosurgery, Henry Ford Health, Detroit, MI, United States; ^2^Department of Neurology, Henry Ford Health, Detroit, MI, United States; ^3^Department of Physics, Oakland University, Rochester, MI, United States

**Keywords:** exosome, stroke, traumatic brain injury (TBI), miRNA, engineered exosome

## Abstract

Traumatic brain injury (TBI) and stroke stand as prominent causes of global disability and mortality. Treatment strategies for stroke and TBI are shifting from targeting neuroprotection toward cell-based neurorestorative strategy, aiming to augment endogenous brain remodeling, which holds considerable promise for the treatment of TBI and stroke. Compelling evidence underscores that the therapeutic effects of cell-based therapy are mediated by the active generation and release of exosomes from administered cells. Exosomes, endosomal derived and nano-sized extracellular vesicles, play a pivotal role in intercellular communication. Thus, we may independently employ exosomes to treat stroke and TBI. Systemic administration of mesenchymal stem cell (MSC) derived exosomes promotes neuroplasticity and neurological functional recovery in preclinical animal models of TBI and stroke. In this mini review, we describe the properties of exosomes and recent exosome-based therapies of TBI and stroke. It is noteworthy that the microRNA cargo within exosomes contributes to their therapeutic effects. Thus, we provide a brief introduction to microRNAs and insight into their key roles in mediating therapeutic effects. With the increasing knowledge of exosomes, researchers have “engineered” exosome microRNA content to amplify their therapeutic benefits. We therefore focus our discussion on the therapeutic benefits of recently employed microRNA-enriched engineered exosomes. We also discuss the current opportunities and challenges in translating exosome-based therapy to clinical applications.

## 1 Introduction

Traumatic brain injury (TBI) and stroke are prominent causes of global disability and mortality, and impose a significant social and economic burden (Hyder et al., 2007; Lackland et al., 2014; Johnson and Griswold, 2017; Kuriakose and Xiao, 2020; Hering and Shetty, 2023). Unfortunately, no effective drugs are available for improving TBI functional recovery, and tPA is the only FDA-approved drug as a treatment for acute ischemic stroke (National Institute of Neurological Disorders and Stroke rt, PA Stroke Study Group, 1995; Hacke et al., 2008). Furthermore, nearly all the phase II/III clinical trials directed towards neuroprotection for TBI and stroke have failed (Xiong et al., 2018).

Recently, attention has shifted towards cell-based neurorestorative strategy, designed to augment endogenous brain remodeling after TBI or stroke. Cell-based therapy, particularly using bone marrow mesenchymal cells (MSCs), has proven to be safe and effective in promoting neuroplasticity and neurorestoration, leading to the improvement of neurological and cognitive function in animal models of TBI and stroke (Chen et al., 2001; Chopp and Li, 2002; Zhang and Chopp, 2009; Cox et al., 2011; Nichols et al., 2013; Bonsack et al., 2020; Pischiutta et al., 2022; Giovannelli et al., 2023). Paracrine effects rather than the direct replacement of injured tissue through stem/progenitor cell differentiation underlie the therapeutic benefits of cell-based therapy (Chopp and Li, 2002; Phinney and Prockop, 2007; Lai et al., 2011; Camussi et al., 2013; Xin et al., 2013a; Pischiutta et al., 2022; Giovannelli et al., 2023; Zhuang et al., 2023). Signaling pathways in the injured brain triggered by paracrine factors released directly or indirectly by MSCs have the potential to promote endogenous neuronal rewiring and enhance angiogenesis and neurogenesis by communication with brain parenchymal cells, and thereby amplify brain remodeling, and ultimately improve functional recovery after injury (Zhang et al., 2019; Pischiutta et al., 2022; Zhuang et al., 2023).

Among these paracrine factors, exosomes, endosome-derived membrane-bound small extracellular vesicles, play a significant role in the intercellular communication (Lai and Breakefield, 2012; Rak, 2013; Lener et al., 2015; Xin et al., 2021; Zhang Y. et al., 2021, 2023; Liu et al., 2022). Exosomes contain proteins, lipids, mRNAs, microRNAs (miRNAs), and long non-coding RNAs. Through the transfer of their molecules via endocytosis or ligand-receptor interactions, exosomes communicate directly or indirectly with endogenous brain cells (Schneider and Simons, 2013). This communication may promote neurorestorative effects and improve functional outcome after TBI or stroke (Xin et al., 2013a, 2014; Kim et al., 2016; Zhang et al., 2017b). Contained within the exosome cargo, miRNAs are of considerable importance in mediating the therapeutic effects of exosomes (Xin et al., 2013a, 2014; Zhang et al., 2019).

In this mini review, we discuss exosome-based treatment as a potential neurorestorative therapy for TBI and stroke. We focus on recent developments of engineered exosome-based treatment, especially specific miRNA enriched exosomes. At the end of this mini review, we discuss the current opportunities and challenges in translation of exosome-based therapy to clinical applications.

## 2 Exosomes properties and physiological functions

Exosomes are membrane-bound vesicles derived from endosomes, typically measuring ~30–150 nm in diameter (Lai and Breakefield, 2012; Rak, 2013). The formation of exosomes is initiated through cellular endocytosis or plasma membrane invagination, leading to the generation of small intracellular bodies called endosomes (Thery et al., 2002). Early endosomes subsequently develop into late endosomes, which contain numerous intraluminal vesicles (ILVs) and is often referred to as a multivesicular body (MVB). Proteins, mRNAs, miRNAs, and DNAs are directly sorted to the MVB from several organelles (Thery et al., 1999). Later, the MVB may either fuse with the lysosome, resulting in the degradation of its contents, or fuse with the plasma membrane, leading to the release of its ILVs into extracellular environment. These vesicles are then called exosomes (van Niel et al., 2006).

The cargos and membrane structure of exosomes are determined by their birth cells under specific physiological and environmental conditions of these cells (Chopp and Zhang, 2015). Under physiological conditions, via transfer of their cargo, exosomes, secreted by brain cells, maintain or regulate brain function (Kalluri and LeBleu, 2020). For example, exosomes derived from neurons maintain the integrity of the blood–brain barrier (BBB) by transferring miRNA-132 to endothelial cells (Xu et al., 2017); oligodendrocyte derived exosomes assist in axonal myelination by delivering myelin proteins proteolipid protein (PLP), 2′3’-cyclic-nucleotide-phosphodiesterase (CNP) and myelin basic protein (MBP) (Kramer-Albers et al., 2007; Fruhbeis et al., 2013a,b); and astrocyte-derived exosomes regulate synaptic plasticity by transporting the miRNA-26 to synapses (Lafourcade et al., 2016).

The mechanisms underlying exosome treatment for brain injury involve complex intercellular communication and the delivery of bioactive molecules to target cells within the injured brain tissue. Exosomes possess anti-inflammatory properties that suppress neuroinflammation in the injured brain (Zhang et al., 2015; Yang Y. et al., 2017; Williams et al., 2020a; Mavroudis et al., 2023). Interacting with immune cells in the brain, such as microglia and astrocytes, exosomes modulate the immune response following injury (Liu et al., 2023). Exosomes exert neuroprotective effects by promoting cell survival and inhibiting apoptosis of injured neurons (Williams et al., 2020a; Zhang et al., 2022). Treatment with exosomes derived from stem cells stimulate angiogenesis (formation of new blood vessels) and neurogenesis (generation of new neurons) in the injured brain (Zhang et al., 2015). In addition, exosomes influence the integrity and permeability of the BBB and play a role in promoting neuronal plasticity by delivering factors that modulate synaptic function and neuronal connectivity (Gao et al., 2018; Williams et al., 2020a; Xia et al., 2022). Overall, exosome therapy for brain injury harnesses a range of molecular mechanisms and pathways to promote neuroprotection, tissue repair, and functional recovery. By capitalizing on the therapeutic potential of exosomes, researchers aim to develop effective treatments for TBI, stroke, and neurodegenerative diseases. A detailed overview of the therapeutic effects and mechanisms underlying naive and engineered exosome treatment for TBI and stroke is provided in following Sections, with a focus on the role of miRNAs in mediating exosome function.

## 3 Exosome-based therapy of stroke and TBI

Paracrine mechanisms underlie the MSC-based therapeutic effects, where MSCs secrete factors that influence endogenous cells (Chopp and Li, 2002; Lai et al., 2011; Camussi et al., 2013; Xin et al., 2013a). Among these paracrine factors, exosomes are critical to cell-based therapeutic actions (Xin et al., 2014; Yang Y. et al., 2017; Xiong et al., 2018; Zhang et al., 2019). Compared to cell-based therapy, exosome-based therapy offers several advantages: (1) exosomes have a superior safety profile due to low immunogenicity and tumorigenesis (El Andaloussi et al., 2013; Xiong et al., 2017); (2) exosome injection has low risk of inducing microvascular embolism due to its nano size (Xin et al., 2014); (3) exosomes can be safely stored without losing function; (4) nano-sized exosomes are capable of crossing the BBB by systemic injection (Chen et al., 2016; Otero-Ortega et al., 2018); (5) the cargo of exosomes can be engineered to amplify their therapeutic effects (Xin et al., 2017b; Chen and Chopp, 2018).

Pioneering research on MSC-exosome-based treatment has been performed in rodent models of stroke and TBI (Xin et al., 2013a; Doeppner et al., 2015; Zhang et al., 2015). In the stroke study, systemic administration of MSC-exosomes (MSC-Exos) in rats 24 h after induction of middle cerebral artery occlusion (MCAO) improved their neurological functional recovery, possibly by enhancing neurite and vascular remodeling and neurogenesis post stroke (Xin et al., 2013a). In the TBI study, MSC-Exos administered intravenously 24 h after TBI promoted neurovascular remodeling, neurogenesis, and sensorimotor and cognitive functional recovery in TBI rats (Zhang et al., 2015). Importantly, MSC-Exos and MSC treatments resulted in equivalent functional improvements in mice post-stroke (Doeppner et al., 2015). Increasing numbers preclinical studies have shown that exosome-based treatment can promote neuroplasticity and neurorestoration by increasing neurite remodeling (Xin et al., 2013a; Otero-Ortega et al., 2017), axonal sprouting (Otero-Ortega et al., 2017; Xin et al., 2017a), synaptogenesis (Xin et al., 2017a), and angiogenesis and neurogenesis (Zhang et al., 2020), ultimately improving functional outcomes in stroke and TBI animals (Zhang et al., 2017b, 2020; Dumbrava et al., 2022; Zhang R. et al., 2023). MSC-Exos also promoted neurological functional recovery and brain tissue remodeling in aged stroke rats (Dumbrava et al., 2022). In addition to neurorestorative effects, exosomes have neuroprotective effects, such as reducing lesion volume in TBI (Zhang W. et al., 2021) or ischemic core (Liu et al., 2021), suppressing cell apoptosis (Zhang W. et al., 2021), and mitigating neuroinflammation (Zhang R. et al., 2023). These preclinical data demonstrated the great therapeutic potential of exosome-based therapy for stroke and TBI.

Following the rodent studies, exosome-based therapy has been further evaluated in large animal models. In a swine model of severe TBI and hemorrhagic shock (HS), early (1 h after shock)-single-dose MSC-Exos-treated animals experienced significantly less functional impairment and faster neurological functional recovery, resulting from reduced brain lesion, inflammation, and apoptosis, as well as promoted neural plasticity (Williams et al., 2020b). In a series of studies using an adult rhesus monkey model with cortical injury, MSC-Exos treatment achieved increased functional recovery of grasp pattern with reduced latency to retrieve a food reward compared to saline treatment (Moore et al., 2019; Go et al., 2020, 2021). These therapeutic effects were attributed to the neuroprotection and neurorestoration effects of MSC-Exos, by reducing neuroinflammation (Go et al., 2020) and suppressing damage to oligodendrocytes to improve the myelin maintenance (Go et al., 2021). This cortical injury model in the monkey is highly related to stroke in human due to the similarity of fine motor function of hand and digits between human and monkey.

In addition to MSCs, exosomes derived from other cell types also induce neurorestoration and improve functional outcomes. For example, neural stem cell (NSC)-derived exosomes reduced inflammatory response, ameliorated brain injury, and improved motor functional recovery in stroke mice (Zhang R. et al., 2023); astrocyte-derived exosomes protected mice and rats against TBI-induced neuronal cell loss/apoptosis and oxidative stress, thereby alleviating functional impairment (Zhang W. et al., 2021). The cargos of exosomes are closely related to their parent cells and mediate their therapeutic effects in stroke and TBI treatment (Zhang et al., 2019). However, the most effective types of cell-derived exosomes for the treatment of stroke or TBI have not yet been determined (Lener et al., 2015).

In summary, the data from rodent and large animal studies suggest that exosome-based therapy provides beneficial therapeutic effects post stroke and TBI, including neurorestoration and neuroprotection (Table 1).

**Table 1:** Selected recent studies of exosomes for treatment of stroke and TBI.

Disease	Animal model	Source of exosome	Therapeutic effects	References
Stroke	Aged rat MCAO	MSCs	Improvement in neurological functional recovery and brain tissue remodeling	Dumbrava et al. (2022)
	Mice MCAO	Neural stem cells (NSCs)	Reduction in inflammation; neuroprotection and improvement in functional recovery	Zhang R. et al. (2023)
	Rat MCAO	Health rat serum	Neuroprotection	Huang et al. (2022)
	Rat MCAO	BMSCs	Reduction in brain infarct area and inflammation; improvement in neurological function	Liu et al. (2021)
	Mice MACO	BMSCs	Reduction in neuronal cell damage; facilitating angiogenesis	Xiao et al. (2023)
	Porcine MACO	hNSCs	Reduction in edema, neuroprotection, functional recovery	Webb et al. (2018)
	Rhesus monkey	MSCs	Fine motor recovery improvement, reduction in neuroinflammation and myelin damage	Go et al. (2020, 2021); Moore et al. (2019)
TBI	Rat weigh-drop	Human adipose mesenchymal stem cell	Suppression in neuroinflammation; improvement in neurogenesis	Chen et al. (2020)
	Rat CCI	Astrocyte	Alleviation in neurobehavior and cognitive deficits; neuroprotection	Zhang W. et al. (2021)
	Rat CCI	MSCs	Improvement in sensorimotor and cognitive function; promotion in angiogenesis and neurogenesis; reduction in cell loss and neuroinflammation	Zhang et al. (2020)
	Swine	hMSCs	Attenuation in neurologic injury, promotion in neural plasticity	Williams et al. (2020b)

## 4 MiRNAs in exosomes are critical to therapeutic effects

As noted, exosomes can alter recipient cells’ function via transfer of their cargo. Systemic administration of liposomes, consisting of lipid components of exosomes but without proteins and genetic materials, had no therapeutic benefits compared to naïve MSC-Exos treatment, indicating that the therapeutic effects of exosomes are derived from the cargo of exosomes (Zhang et al., 2017b). Among the exosomal cargo, miRNAs play a substantial role in therapeutic effects of exosomes.

MiRNAs are small (22 nucleotides in length), evolutionary conserved, non-coding RNA molecules (Macfarlane and Murphy, 2010; O'Brien et al., 2018). The biogenesis of most miRNAs is initiated from the transcription of a DNA sequence, and transcribed primary miRNAs are subsequently processed into precursor miRNAs and mature miRNAs (O'Brien et al., 2018). Each miRNA is a master molecular switch, which regulates the gene expression of hundreds of mRNAs at post-transcriptional level by binding to complementary sequences on mRNA, consequently causes the cleavage of mRNAs or translation repression (Cai et al., 2009; Macfarlane and Murphy, 2010).

Through genetic approaches, many studies have demonstrated that miRNAs contribute to the therapeutic effect of exosomes (Mateescu et al., 2017). Dicer, a ribonuclease, is involved in the production of mature miRNAs. Conditional knockout of Dicer (Dicer/Cko) in adult neural progenitor cells (NPCs) substantially reduced cellular miRNAs, and impaired neurogenesis and cognitive function in Dicer/Cko mice, while administration of cerebral endothelial-derived exosomes carrying mature miRNAs restored neurogenesis and cognitive function (Zhang, R. L. et al., 2017). Another example is Argonaute 2 (Ago2), a primary miRNA machinery protein required for packaging miRNAs into exosomes and performing activities in the recipient cells (Gibbings et al., 2009). MSC-Exos promoted the axonal growth for cortical neurons while attenuation of Ago2 protein in MSC-Exos abolished their effect on axonal growth (Zhang et al., 2017a). Attenuation of Ago2 protein in MSCs reduces miRNAs in MSC-Exos and reduces exosome treatment-induced beneficial effects in TBI recovery (Zhang Y. et al., 2023). Collectively, these studies indicate that therapeutic benefits of exosomes are substantially attributed to their miRNA cargo.

## 5 Engineered exosomes to amplify therapeutic benefits

Since miRNAs significantly contribute to the exosome’s therapeutic benefits, the use of engineered exosomes with enriched specific miRNAs for the treatment of stroke and TBI is under active investigation in order to amplify their therapeutic benefits. Generally, designing miRNA enriched engineered exosomes begins with brain miRNA profiling after stroke or TBI (Deng et al., 2019; Zhuang et al., 2023). Dysregulation of miRNA expression is related to neurodegenerative diseases and brain injuries, including stroke and TBI (Pan et al., 2017; Atif and Hicks, 2019). MiRNA microarray and next-generation sequencing platform are employed to quantify miRNA expression profiles in brain tissue, blood, and cerebrospinal fluid of animal models and humans with stroke or TBI (Redell et al., 2010; Di Pietro et al., 2017; Pan et al., 2017). After comparing these miRNA profiles with those from healthy animals or humans, researchers apply bioinformatic analysis to identify specific microRNAs that play crucial roles in neuroprotection, neuroregeneration, and anti-inflammatory responses, and anticipate the candidate gene targets of these miRNAs to determine if they may be a target for stroke or TBI treatment (Zhuang et al., 2023). Then, researchers tailor the composition of engineered exosomes to carry these microRNAs for precise targeting of pathways involved in TBI and stroke recovery by altering the genetic character of cells, e.g., by transfection or electroporation miRNA mimics (agomir) / inhibitors (antagomir), finally generating specific miRNA enriched/decreased engineered exosomes (Wen, 2016; Sun et al., 2018). Recently published research about miRNAs enriched exosomes for the treatment of brain diseases is listed in Table 2.

**Table 2:** Studies of miRNA-enriched exosomes for treatment of neural injuries.

Disease	Animal model	Source of exosome	miRNA enriched	Amplified therapeutic effects (compared to naïve exosomes)	References
Stroke	Rat MCAO	MSCs	miRNA-17-92	Enhancement of axon-myelin remodeling and functional recovery	Xin et al. (2021)
	Rat MCAO	MSCs	miRNA-17-92	Increasement of neural plasticity and functional recovery	Xin et al. (2017a)
	Rat MCAO	MSCs	miRNA-133b	Improvement of functional recovery and brain plasticity	Xin et al. (2017b)
	Mice	Endothelial progenitor cells (EPCs)	miRNA-126	Attenuation of acute injury and promotion of functional recovery	Wang et al. (2020)
	Rat MCAO	MSCs	miRNA-145	Decreasing infarct area in MCAO rat	Zhou et al. (2022)
	Mice MCAO	Neural progenitor cells (NPCs) and EPCs	miRNA-210 miRNA-126	Reduction of cell apoptosis and ROS production; promotion of neurite outgrowth	Xu et al. (2023)
	Rat MCAO	Adipos-derived stem cells (ADSCs)	miRNA-30d-5p	Neuproprotection; Reduction of neuroinflammation	Jiang et al. (2018)
	Mice MCAO	BMSCs	MiRNA-138-5p	Reduction of neurological impairment and inflammatory response	Deng et al. (2019)
TBI	Rat CCI	MSCs	miRNA-17-92	Reduction of neuroinflammation; neuroprotection; enhancement of angiogenesis and neurogenesis	Zhang Y. et al. (2021)
	Rat CCT	MSCs	miRNA-124	Reduction of neuroinflammation; improvement of neurogenesis and functional recovery	Yang et al. (2019)
	Mice rmTBI	Microglia	miRNA-124-3p	Alleviation of neurodegeneration; improvement of cognitive outcome	Ge et al. (2020)
	Rat CCI	BMSCs	miRNA-124-3p	Neuroprotection; improvement of cognitive outcome	Zhuang et al. (2023)
	Mice rmTBI	microglial	miRNA-124-3p	Inhibition of neuronal autophagy; reduction of never injury	Li et al. (2019)
	Rat ICH	MSCs	miRNA-133b	Neuroprotection	Shen et al. (2018)
Intracerebral hemorrhage (ICH)	Rat ICH	BMSCs	miRNA-146a-5p	Reduction of neuronal apoptosis and inflammation	Duan et al. (2020)
ICH	Mice	BMSCs	MiRNA-193b-3p	Attenuation of neuroinflammation and neurobehavioral impairments	Lai et al. (2020)
Subarachnoid hemorrhage (SAH)	Rat SAH	MSCs	miR-21-5p	Neuroprotection	Gao et al. (2020)

The therapeutic potential of miRNAs enriched engineered exosomes derived from MSCs in stroke rodent model was first documented in 2013 (Xin et al., 2013b). In 2017, three engineered exosomes with elevated miRNAs were developed and provided an increased therapeutic effect on neurological recovery compared with naïve exosomes (Xin et al., 2017a,b; Yang J. et al., 2017). Systemic administration of MSC-Exo-17-92 in a rat MCAO model at 24 h after induction of stroke significantly improved sensorimotor functional recovery and enhanced neurogenesis, neurite plasticity, and oligodendrogenesis compared to naïve exosome treatment. This enhanced therapeutic benefit was attributed to downregulation of phosphatase and tension homolog (PTEN), and subsequent activation of the P13K/phosphorylated mammalian target of rapamycin (mTOR) signaling pathways targeted by the miRNA-17-92 cluster (Xin et al., 2017a). In another rodent study, MSC-Exo-133b treatment increased secondary release of exosomes from astrocytes and promoted neurite outgrowth and plasticity and functional recovery in stroke rats (Xin et al., 2017b). MSC-Exo with elevated miRNA-124 (MSC-Exo-124) with rabies virus glycoprotein (RVG) fused to the exosomal protein lysosome-associated membrane glycoprotein 2b (Lamp2b) delivered miRNA-124 more efficiently to the infarct site, and further promoted cortical neurogenesis in a mouse model of ischemic stroke (Yang J. et al., 2017).

Currently, select exosomal miRNAs from different cell lines have been verified to mediate neurorestorative function via promoting neurogenesis, angiogenesis, axonal remodeling, and neuronal plasticity (Wang et al., 2020; Nasirishargh et al., 2021). For example, miRNA-126 can regulate vascular integrity and promote angiogenesis via activating vascular endothelial growth factor receptor 2 (VEGFR2) (Wu et al., 2016). In a diabetic stroke mouse model, miRNA-126 enriched exosomes derived from endothelial progenitor cells (EPC-Exo-126) were rapidly taken up by brain neurons, endothelial cells, astrocytes, and microglia in the peri-infarct area after treatment, which more effectively promoted neurogenesis and angiogenesis than the naïve EPC-Exo, and enhanced functional recovery post-stroke (Wang et al., 2020). Engineered exosomes have also been applied to TBI treatment via boosting neurorestoration. Intravenous administration of MSC-Exo-17-92 to rat after 24 h of TBI significantly increased neurogenesis and angiogenesis in the hippocampus, which promoted neuronal functional recovery (Zhang Y. et al., 2021); MSC-Exo-124 elevated hippocampus neuronal proliferation and differentiation after TBI in rats, possibly by inhibiting the inflammation via regulating the TLR4 pathway (Yang et al., 2019).

Many exosomal miRNAs also play a neuroprotective role in stroke and TBI (Panaro et al., 2020). MiR-124, the most highly expressed miRNA in the central nervous system (CNS), is significantly altered in the acute, sub-acute and chronic phase post-TBI (Li et al., 2019; Ge et al., 2020; Zhuang et al., 2023). Microglia derived Exo-124-3p (Microglia-Exo-124-3p) alleviated neurodegeneration by reducing neurite branch loss and inhibiting β-amyloid (Aβ) by targeting the Rela/ApoE signaling pathway in a mouse repetitive (r)TBI (Ge et al., 2020); MSCs-derived Exo-124-3p (MSCs-Exo-124-3p) reduced neuronal cell death and minimized the lesion volume post-TBI in rats likely via attenuating posttraumatic glutamate-mediated excitotoxicity by downregulating p38MAPK expression (Zhuang et al., 2023). Additionally, engineered exosomes, e.g., MSC-Exo enriched with miRNA-138-5p (MSC-Exo-138-5p), miRNA-145, (MSC-Exo-145), miRNA-21-5p (MSC-Exo-21-5p), and adipose-derived stem (ADSCs) derived exosomes enriched with miRNA-30d-5p (ADSC-Exo-30d-5p) provided neuroprotection after neural injury (Jiang et al., 2018; Deng et al., 2019; Gao et al., 2020; Zhou et al., 2022). For example, the treatment of MSC-Exo-138-5p reduced apoptosis of astrocytes in ischemic stroke mice (Deng et al., 2019); ADSC-Exo-30d-5p significantly reduced infarct volume and neuronal apoptosis in stroke rats (Jiang et al., 2018).

Chronic neuroinflammation aggravates neurodegeneration and impedes neuronal repair after stroke and TBI (Xiong et al., 2018). Several miRNA-enriched exosomes are designed to regulate neuroinflammation. MSC-Exo-145 promoted the conversion of microglia from a pro-inflammatory M1 to an anti-inflammatory M2 phenotype *in vitro*, possibly by downregulating FOXO1 (Zhou et al., 2022). ADSC-Exo-30d-5p regulated the neuroinflammation by suppressing autophagy, M1 polarization of microglial cells, and inflammatory cytokines *in vitro* and *in vivo*, which reduced infarct size in stroke rats (Jiang et al., 2018). Moreover, MSC-Exo- and microglia-Exo-124 treatment promoted M2 polarization of microglia, and inhibited autophagy and inflammatory cytokines in a TBI rodent model (Li et al., 2019; Yang et al., 2019).

Looking forward, miRNA-enriched engineered exosomes could be further modulated to enhance treatment efficiency and amplify therapeutic effects. For example, miRNA-enriched exosomes could incorporate targeting strategies to enable site-specific drug delivery; engineered exosomes could carry multiple miRNAs to target different injury mechanisms after neural injury.

In summary, these preclinical data suggest that modulating miRNAs content of exosomes is a feasible and promising means to amplify the therapeutic effects of exosomes for the treatment of stroke and TBI, as well as degenerative diseases.

## 6 Discussion

Exosomes play a substantial role in intercellular communication. By transferring their cargo, exosomes can induce neurorestorative and neuroprotective effects via regulating genes and protein expression in target cells or tissues post-injury. Collectively, data from preclinical studies indicate that exosome-based therapy could promote neuroplasticity, reduce impairments, and accelerate functional recovery in animal models of stroke or TBI. Although the mechanisms that underlie the benefits are not fully understood, exosome-based approach as potential therapy for stroke and TBI is under active investigation (Zhang et al., 2019). Engineered exosomes with modified cargos are designed to amplify the therapeutic efficacy, in which specific miRNAs enriched engineered exosomes stand in the spotlight and provide better therapeutic efficacy than naïve exosomes.

Although the data from preclinical proof-concept studies are promising, there are several challenges in the translation of exosome-based therapy to clinical application. Firstly, a greater understanding of the mechanism of exosomes action is needed. This will secure the safety of exosome-based therapy and be the foundation of designed engineered exosomes. Secondly, standardization of exosome isolation, scale-up production, characterization, and cargo analysis methods are necessary for human clinical trials (Lener et al., 2015). Thirdly, the determination of optimal dose, therapeutic windows and cell sources of exosomes are crucial for successful clinical translation (Xiong et al., 2017). Performance of safety studies for using exosomes must be fully investigated, such as oncogenic potential studies (Jayaraman et al., 2017; Latchana et al., 2017; Zhang H. et al., 2017). It is because many miRNAs also are closely linked with oncogenesis (Chen et al., 2013; Zhang H. et al., 2017). Therefore, studies should be performed to ensure that the restorative exosomes would not further induce tumor growth. Collectively, further preclinical studies of cell source selection, scale-up production, safety, dose–response, time window, administration routes, cargo analyses, and mechanisms of naive and engineered exosomes are required before effective and safe clinical translation.

## Author contributions

LC: Conceptualization, Writing – original draft, Writing – review & editing. YX: Conceptualization, Funding acquisition, Supervision, Writing – review & editing. MC: Conceptualization, Funding acquisition, Supervision, Writing – review & editing. YZ: Conceptualization, Funding acquisition, Supervision, Writing – review & editing.
